# Zcchc11 Uridylates Mature miRNAs to Enhance Neonatal IGF-1 Expression, Growth, and Survival

**DOI:** 10.1371/journal.pgen.1003105

**Published:** 2012-11-29

**Authors:** Matthew R. Jones, Matthew T. Blahna, Elyse Kozlowski, Kori Y. Matsuura, Joseph D. Ferrari, Samantha A. Morris, John T. Powers, George Q. Daley, Lee J. Quinton, Joseph P. Mizgerd

**Affiliations:** 1 Pulmonary Center, Boston University School of Medicine, Boston, Massachusetts, United States of America; 2 Molecular and Integrative Physiological Sciences Program, Harvard School of Public Health, Boston, Massachusetts, United States of America; 3 Genetics and Genomics Graduate Program, Boston University School of Medicine, Boston, Massachusetts, United States of America; 4 Stem Cell Program, Children's Hospital Boston, Department of Biological Chemistry and Molecular Pharmacology, Harvard Medical School, Harvard Stem Cell Institute, Boston, Massachusetts, United States of America; 5 Departments of Medicine, Microbiology, and Biochemistry, Boston University School of Medicine, Boston, Massachusetts, United States of America; Cincinnati Children's Hospital Medical Center, United States of America

## Abstract

The Zcchc11 enzyme is implicated in microRNA (miRNA) regulation. It can uridylate let-7 precursors to decrease quantities of the mature miRNA in embryonic stem cell lines, suggested to mediate stem cell maintenance. It can uridylate mature miR-26 to relieve silencing activity without impacting miRNA content in cancer cell lines, suggested to mediate cytokine and growth factor expression. Broader roles of Zcchc11 in shaping or remodeling the miRNome or in directing biological or physiological processes remain entirely speculative. We generated Zcchc11-deficient mice to address these knowledge gaps. Zcchc11 deficiency had no impact on embryogenesis or fetal development, but it significantly decreased survival and growth immediately following birth, indicating a role for this enzyme in early postnatal fitness. Deep sequencing of small RNAs from neonatal livers revealed roles of this enzyme in miRNA sequence diversity. Zcchc11 deficiency diminished the lengths and terminal uridine frequencies for diverse mature miRNAs, but it had no influence on the quantities of any miRNAs. The expression of IGF-1, a liver-derived protein essential to early growth and survival, was enhanced by Zcchc11 expression *in vitro*, and miRNA silencing of IGF-1 was alleviated by uridylation events observed to be Zcchc11-dependent in the neonatal liver. In neonatal mice, Zcchc11 deficiency significantly decreased IGF-1 mRNA in the liver and IGF-1 protein in the blood. We conclude that the Zcchc11-mediated terminal uridylation of mature miRNAs is pervasive and physiologically significant, especially important in the neonatal period for fostering IGF-1 expression and enhancing postnatal growth and survival. We propose that the miRNA 3′ terminus is a regulatory node upon which multiple enzymes converge to direct silencing activity and tune gene expression.

## Author Summary

MicroRNAs (miRNAs) are molecules that regulate gene expression, usually serving silencing functions. Mechanisms regulating miRNAs are poorly understood. In test tube experiments, the enzyme Zcchc11 adds uridines to the ends of miRNAs and their precursors, with uridyation of miRNA precursors decreasing the quantities of mature miRNAs and uridylation of mature miRNAs decreasing their silencing activity. Whether, when, and to what effect Zcchc11 alters miRNA in living animals has never previously been reported. To understand functions of Zcchc11 in integrative biology, we generated mice deficient in Zcchc11. Mutant mice were born normally, but some died soon after birth and survivors grew poorly. No miRNA quantities were changed in tissues sampled from these mice, but mature miRNAs were less likely to have additional uridines on their ends. Some miRNAs that were uridylated by Zcchc11 targeted a critical growth factor known as insulin-like growth factor 1 (IGF-1), but they did so less effectively when uridylated. Zcchc11-deficient mice had decreased amounts of IGF-1 in the liver and blood. These data reveal that Zcchc11 is an important enzyme in living animals for uridylating mature miRNAs, enhancing IGF-1 expression, and promoting neonatal growth and survival, suggesting a novel mode of gene regulation that is biologically significant.

## Introduction

Non-canonical poly(A) polymerases (PAPs) comprise a family of enzymes highly conserved across Eukaryota and capable of catalyzing the template-independent transfer of uridines and adenines onto single-stranded RNA substrates [1], [2]. Several non-canonical PAPs, including the uridyltransferase Zcchc11 (PAPD3/TUT4), can mediate 3′ terminal nucleotide additions to mature miRNA [3], [4], [5]. The uridylation or adenylation of mature miRNAs does not impact miRNA quantity, but instead limits miRNA silencing of select, targeted transcripts [3], [5]. By other means, Zcchc11 can regulate quantities of mature miRNA. In mouse embryonic stem cell lines, Zcchc11 recognizes complexes of Lin28 and pre-let-7 and adds an oligouridine tail to the 3′ terminus of the pre-miRNA, preventing maturation and/or enhancing degradation of the precursor [6], [7]. Knockdown of Zcchc11 or Lin28 in stem cell lines increases mature let-7 and decreases pluripotency markers [6], [8], [9]. Both of these models propose that uridylation by Zcchc11 circumvents miRNA-mediated transcript silencing, but they invoke disparate mechanisms. While not mutually exclusive, they have never been concurrently examined, and each has been demonstrated only in reductionist cell line systems focusing on small subsets of miRNAs. The degree to which miRNA quantity and/or sequence diversity may be remodeled by Zcchc11 (or any non-canonical PAP) and the roles of Zcchc11 in integrated biological systems remain speculative and represent major knowledge gaps.

## Results

### Zcchc11 enhances the growth and survival of young mice

**Figure 1:**
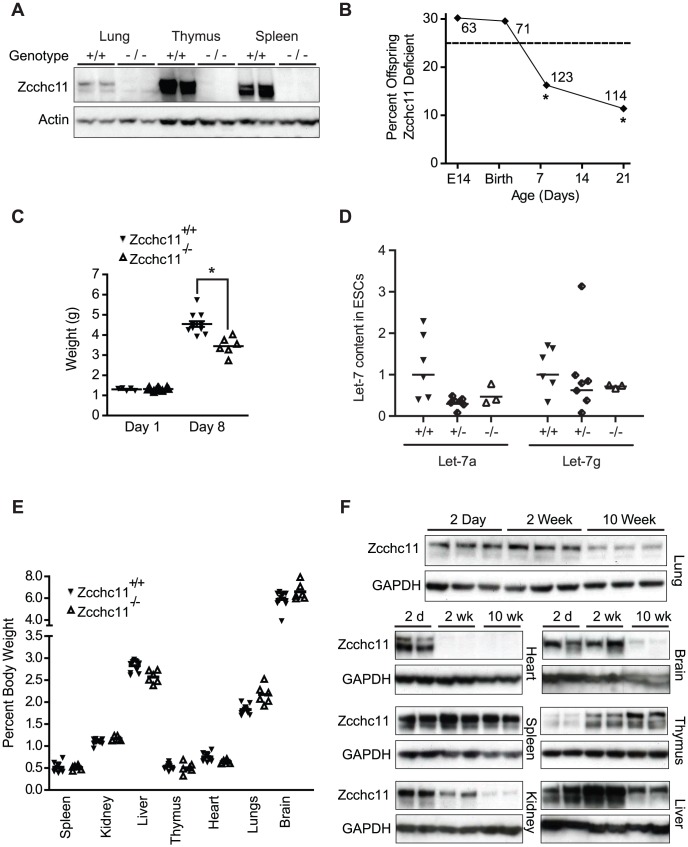
Zcchc11 enhances growth and fitness through the perinatal period. (A) Immunoblots of organs from C57BL/6 and Zcchc11^−/−^ mice show deletion of Zcchc11 protein. (B) Fraction of homozygous Zcchc11-deficient offspring from Zcchc11^+/−^ parents at day E14, P1, P8 and P21, indicating decreased survival by day 8. *p<0.05 *vs.* 0.25 by Chi-squared test, N as indicated. (C) Body weights of Zcchc11^+/+^ and Zcchc11^−/−^ littermates at day 1 and 8 (*p<0.05), showing poor growth in mutants. (D) Let-7 content in primary embryonic stem cell (ESC) cultures that were wild type (+/+), heterozygous (+/−), or deficient (−/−) in Zcchc11 expression, showing no significant effects of genotype (by two-way ANOVA). (E) Proportion of organ weight to body weight in 8-day-old C57BL/6 and Zcchc11^−/−^ mice, showing no difference between genotypes across tissues and suggesting a system-wide growth defect rather than organ-specific effects. (F) Age-dependent expression of Zcchc11, as shown by immunoblots of tissues from C57BL/6 mice at 2 days, 2 weeks, and 10 weeks of age, reveals strongest expression in most organs at young ages. GAPDH is provided as a loading control.

We derived a line of mutant mice from embryonic stem cells carrying a gene-trap insertion in the fourth intron of the 31 exon *Zcchc11* gene [10]. This mutation (Figure S1A) was upstream of all known protein domains and effectively ablated Zcchc11 expression (Figure 1A and Figure S1B). Because Zcchc11 is necessary to limit let-7 in embryonic stem cell lines [6], [7], we anticipated that its loss might be incompatible with development and viability *in utero*. Surprisingly, there was no evidence of decreased fitness through gestation, with offspring from heterozygous parents born in Hardy-Weinberg equilibrium (Figure 1B) and displaying normal morphology and weights (Figure 1C). Furthermore, we observed no increase in let-7 content in Zcchc11-deficient embryonic stem cells compared to embryonic stem cells that were wild type or heterozygous for Zcchc11 (Figure 1D). Thus, Zcchc11-deficient embryonic stem cells were not disadvantaged, and Zcchc11 is dispensable for embryonic stem cell maintenance.

By post-partum day 8, we observed approximately 50% mortality in the Zcchc11-deficient mice (Figure 1B). While initially similar in size, Zcchc11-deficient pups grew more slowly than wild type littermates in the first week (Figure 1C). Most organs were proportionately affected, suggesting systemic effects (Figure 1E). Early bodyweight differences were sustained for months (Figure S1C), but otherwise those mutants surviving beyond the perinatal period displayed no defects in lifespan or gross or histologic morphology (Figure S1D). In contrast to the more restricted tissue expression observed in adult mice [3], Zcchc11 expression was found to be nearly ubiquitous in the first days after birth with protein levels in all organs examined except the thymus decreasing over time (Figure 1F). These findings highlight the neonatal period as a time when Zcchc11 is fundamental to mouse growth and development.

### Zcchc11 does not affect mature miRNA quantities

To address the predominant functions for Zcchc11 in miRNA biology *in vivo*, we used deep sequencing of small RNAs to simultaneously determine mature miRNA quantity and end-modification in an unbiased and comprehensive fashion. We focused our deep sequencing efforts on the liver because it has a relatively homogenous population of cells, Zcchc11 is strongly expressed in this tissue in young mice, and there was precedent for miRNA terminal modifications in the liver [4]. Libraries of small RNAs were constructed from 3 different paired sets of 8-day-old livers from *Zcchc11* ^+/+^ or *Zcchc11* ^−/−^ sex- and littermate-matched mice. More than 85 million sequences were obtained, which exhibited a total of >75% alignment to miRBase (Table S1).

**Figure 2:**
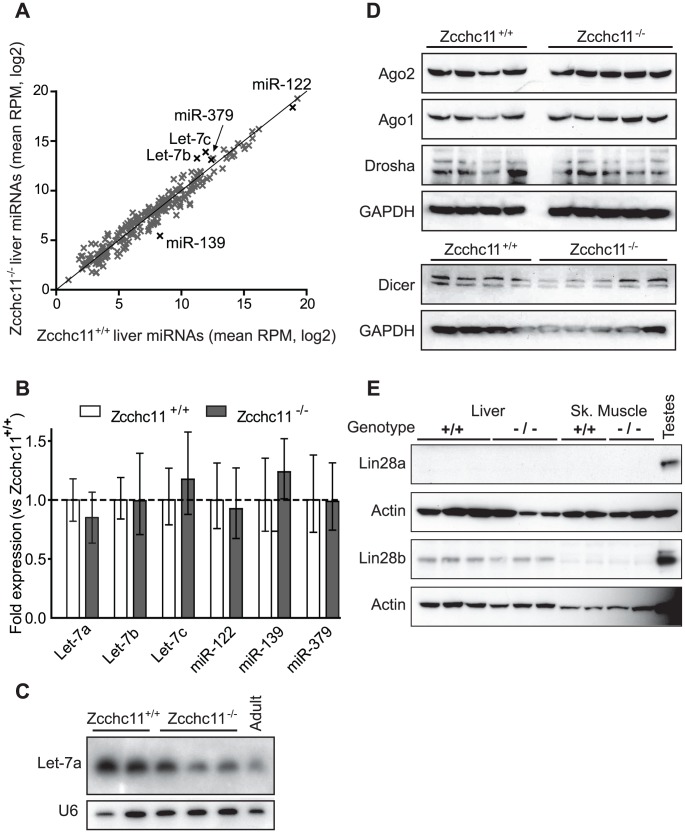
Zcchc11 deficiency does not affect quantities of mature miRNAs or miRNA–related proteins in the liver. Three deep sequencing libraries were created from livers of sex- and littermate-matched 8-day-old Zcchc11^+/+^ or Zcchc11^−/−^ mice. (A) Mature miRNA content, expressed as reads per million (RPM) was compared for wild type and Zcchc11-deficient livers (correlation coefficient, r = 0.975). (B) Quantitative RT-PCR was used to measure the expression of several miRNAs including some implicated in Zcchc11 pre-miRNA uridylation (Let-7), those highly expressed in the livers (miR-122), and those showing trends towards change in the deep sequencing data (miR-139 and miR-379), revealing no difference between genotypes. (C) As another approach to examining Let-7 content, Let-7a in the livers of 8-day old mice was measured by Northern blotting, and did not increase due to Zcchc11 deficiency. An adult wild type mouse was also included for comparison. (D) Immunoblots for RISC-related proteins in tissue homogenates were prepared from the livers of 8 day-old mice, showing no differences due to genotype. (E) Immunoblots for Lin-28 family members in the livers and skeletal muscles of 8 day old Zcchc11^+/+^ and Zcchc11^−/−^ mice revealed no effects of Zcchc11 deficiency. Actin was measured as a loading control. For all blots, each lane represents the RNA or protein from a separate individual of the indicated age and genotype.

Since Zcchc11-mediated uridylation of let-7 precursors in embryonic stem cells diminishes mature let-7 abundance [6], [7], we expected to observe increased quantities of these and perhaps other miRNAs in the Zcchc11-deficient livers. Instead, all detectable miRNAs exhibited a strong correlation in quantity between wild type and Zcchc11-deficient mice (Figure 2A). There were no significant differences due to genotype in the content of any mature miRNA in the liver. Cross-method validation using qRT-PCR analyses of representative miRNAs of interest confirmed that levels of let-7a, let-7b, let-7c, miR-122, miR-139, and miR-379 were equivalent (Figure 2B). A third independent method, northern blotting, also confirmed no increase in let-7a due to Zcchc11 deficiency (Figure 2C). Consistent with this observation, there was no aberrant expression of core miRNA machinery, including Drosha, Dicer, and Argonaute in Zcchc11-deficient livers (Figure 2D). Because mature miRNA levels were unchanged, we considered that Lin28 proteins might be absent from these tissues. While Lin28a was undetectable, Lin28b was present in young mouse livers and was unaffected by Zcchc11 deficiency (Figure 2E). This may be relevant, since Lin28a is more specifically tied to Zcchc11 regulation of let-7, relating to cytoplasmic localization [11]. Altogether, the present data suggest that the high level of Zcchc11 expression in the young liver does not influence mature miRNA abundance, including let-7 family members.

### Zcchc11 mediates mature miRNA lengths and terminal uridine frequencies

**Figure 3:**
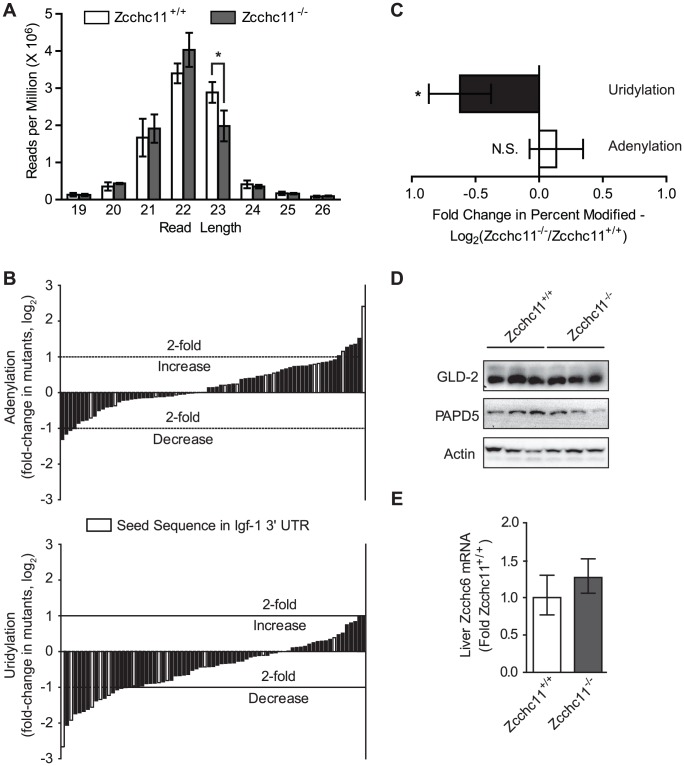
Zcchc11 is essential for the uridylation of miRNAs. The miRNA libraries from Zcchc11-defficent and wild type livers at 8 days of age were analyzed for differences in sequence diversity. (A) Histogram of the number of sequences of a given read length across the libraries, showing fewer 23 nucleotide-long reads in the mutant samples. *p<0.05 by two-way ANOVA with Bonferroni *post hoc* test. (B) End modifications, identified as any nucleotide one base beyond the length of the miRNA published in miRBase, were quantified for each library. Most miRNAs ending in uridine in the control mice did so less frequently in Zcchc11-deficient mice. The mean of the fold change in the percent of sequences adenylated or uridylated between the knockout and wild type were graphed as waterfall plots, with every bar representing the geometric mean of (percent of miR-X sequences modified in the mutants)/(percent of miR-X sequences modified in the WT) for the three separate libraries. Only miRNAs with >10,000 reads and >0.1% sequences end-modified in the wild type libraries were included. Data were expressed as fold-change in Zcchc11^−/−^ mice compared to Zcchc11^+/+^ mice. Light grey bars indicate miRNAs with a seed sequence complementary to a portion of the IGF-1 3′ UTR. (C) Average fold change across all miRNA species shown in Figure 3B, showing that uridines but not adenines were less frequent at miRNA termini due to Zcchc11 deficiency. Error bars indicate 95% c.i. *p<0.05 *vs.* 0 by student's t-test. (D) Immunoblots for the indicated non-canonical poly(A) polymerases in tissue homogenates prepared from the livers of 8 day-old mice, showing no effect of genotype. (E) qRT-PCR for Zcchc6 expression in the livers of 8 day-old mice, showing no effect of genotype.

In addition to precursor modification, Zcchc11 family members are capable of uridylating or adenylating the 3′ termini of mature miRNAs [3], [4], [5], [12]_ENREF_13. While the general pattern of small RNA read lengths from our deep sequence libraries was similar between genotypes, with sequence lengths of 21–23 nucleotides predominating, there were significantly fewer 23 nucleotide-long sequences in the livers of the Zcchc11-deficient mice (Figure 3A). To identify potential enzymatic additions by Zcchc11, aligned sequences were interrogated at the position 1 nucleotide beyond the 3′ terminal residue listed in miRBase [13], [14]. Potential uridylation and adenylation events, included if present at levels of 1 or more sequence per 1,000 reads, were identified for many miRNA species in the livers of wild type mice (Figure S2). Some of these highly modified species, such as miR-26b and miR-122, match those identified previously [4],[5], while others represent novel modification targets. We compared the levels of terminal adenines and uridines in mutant mice for each of the miRNAs that were so modified in the livers of wild type mice. A waterfall plot depicting the changes in terminal adenines between the livers of wild type and Zcchc11-deficient mice revealed a balanced distribution with a mean centered around 0 (Figure 3B–3C). Conversely, the distribution of terminal uridines was significantly skewed, with most of the sequences bearing terminal uridines occurring less frequently in the absence of Zcchc11, suggesting that miRNA uridylation was broadly decreased in Zcchc11-deficient livers (Figure 3B–3C). The expression of other non-canonical PAPs, including PAPD5, GLD-2, and Zcchc6, were unchanged in the livers of Zcchc11 deficient mice (Figure 3D–3E).

Terminal uridines that are 3′ of the expected mature miRNA sequence and dependent on Zcchc11 most likely represent enzymatic additions, but they could also arise from alternative processing of the pre-miRNA. To complement the above analyses, we analyzed miRNAs with terminal uridines that were not genomically encoded and therefore could only result from enzymatic addition, referred to as unambiguous uridylation events. For miRNAs observed in every library from both genotypes, 179 different species showed evidence of unambiguous uridine additions in all 3 wild type libraries, whereas only 118 did so across mutant libraries (p<0.001, χ**^2^** test). This analysis supports the conclusions of the more comprehensive analyses above (Figure 3), together indicating definitively that Zcchc11 deficiency decreases the terminal uridylation of mature miRNAs. These results provide the first compelling evidence, to our knowledge, that Zcchc11 plays a specific and essential role in the length and uridylation of a broad swath of mature miRNAs *in vivo*.

### Zcchc11 uridyltransferase activity contributes to IGF-1 expression

**Figure 4:**
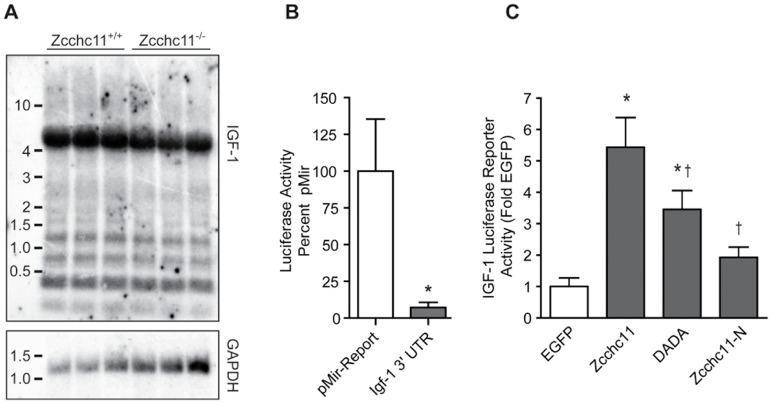
Zcchc11 stabilizes the IGF-1 3′ UTR. (A) Northern blotting was used to identify the predominant IGF-1 isoform expressed in the livers of wild type and Zcchc11-deficient mice at 8 days old. (B) The 3′ UTR from this isoform, cloned onto the end of a firefly luciferase reporter and co-transfected, along with a *Renilla* Luciferase containing a minimal promoter for normalization, decreased reporter expression in H1299 cells. (C) These same constructs were transfected in H1299 cells along with plasmids encoding EGFP, Zcchc11, catalytically inactive Zcchc11 (DADA), or the N-terminal half of Zcchc11. The full-length Zcchc11 increased expression of the IGF-1 3′ UTR reporter, which was significantly inhibited by selective mutation of the catalytic domain or complete deletion of the C terminal half. *p<0.05 *vs.* EGFP *†*p<0.05 *vs* Zcchc11 by one-way ANOVA.

Of the miRNAs ending in uridine more frequently in wild type livers compared to mutants, many were predicted to target IGF-1 (Figure 3B), a growth factor which is liver-derived and essential to early growth and survival in mammals [15], [16]. The IGF-1 3′-UTR is highly polymorphic [17]. We found that an approximately 6.5 kb 3′-UTR was predominant in 8 day old mouse livers (Figure 4A). We cloned this isoform from the livers of C57BL/6 mice and incorporated it into a reporter plasmid to assess the effect of Zcchc11 on the IGF-1 3′-UTR. Addition of this UTR significantly decreased reporter expression *vs.* the coding region alone (Figure 4B), as would be expected for a long 3′-UTR that likely contains many negative regulatory elements. To test the effect of Zcchc11 expression on the IGF-1 3′-UTR, this reporter was co-transfected into cells along with enhanced GFP (EGFP, control), wild type Zcchc11, or Zcchc11 mutants lacking enzymatic activity. Overexpression of wild type Zcchc11 significantly increased levels of the IGF-1 3′-UTR reporter (Figure 4C), indicating that Zcchc11 facilitates IGF-1 expression through its 3′-UTR. Importantly, a catalytically null mutant Zcchc11, in which 2 aspartic acid residues necessary for uridyltransferase activity were changed to alanines [3], was significantly less capable of amplifying the IGF-1 reporter (Figure 4C). Moreover, an N-terminal deletion mutant of Zcchc11 lacking the C-terminal half of the protein and devoid of the uridyltransferase domain, PAP-associated domains, and RNA-binding zinc knuckles was completely incapable of altering IGF-1 expression (Figure 4C). Thus, Zcchc11 can enhance IGF-1 expression through a uridyltransferase-dependent mechanism.

### MicroRNA silencing of IGF-1 is inhibited by terminal uridine addition

**Figure 5:**
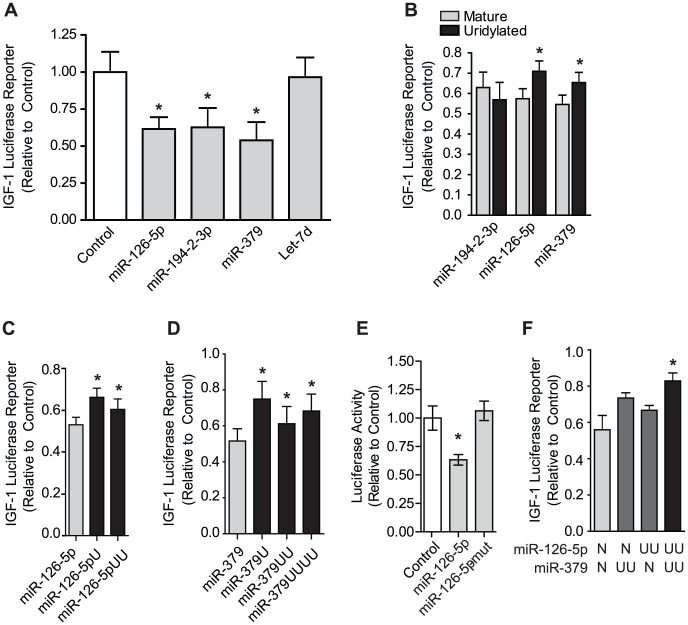
Uridylation of select miRNAs relieves silencing of the IGF-1 3′ UTR. To assess miRNA targeting of IGF-1 the firefly luciferase reporter carrying the IGF-1 3′-UTR and control *Renilla* Luciferase (for normalization) were co-transfected into H1299 cells with various miRNA mimetics. (A) Mimetics for miR-126-5p, miR-194-2-3p and miR-379 effectively targeted the IGF-1 3′-UTR, while Let-7d did not. *p<0.05 *vs*. control, N = 3, by one-way ANOVA. (B) Mimetics of miR-194-2-3p, miR-126-5p, and miR-379 with or without 2 3′ uridine residues were used to assess the impact of uridylation on the effect of silencing by these miRNAs, revealing that uridylation diminished silencing by miR-126-5p and miR-379 but not miR-194-2-3p. *p<0.05 *vs.* mature by two-way ANOVA by student's t-test, N = 4. (C–D) miR-126-5p or miR-379 mimetics bearing 0, 1, 2, or 4 terminal uridines show a maximal effect on miRNA silencing by a single uridine addition. Sequences reflect those identified in sequencing libraries. *p<0.05 *vs.* control by one-way ANOVA, N = 4. (E) Unlike terminal uridylation, mutation of two bases in the seed sequence of miR-126-5p completely reversed silencing of the IGF-1 3′-UTR. (F) Non-uridylated or dual-uridylated mimetics of miR-126-5p and miR-379 illustrate the combinatorial role of multiple uridylated miRNAs targeting the same transcript. *p<0.05 *vs* control by one-way ANOVA, N = 4.

Of the multiple miRNAs which had terminal uridines that required Zcchc11 in our deep sequencing datasets and were predicted to target the 3′ UTR of IGF-1 (Figure 3B), we examined the ability of 4 (miR-126-5p, miR-194-2-3p, miR-379 and Let-7d) to suppress IGF-1 expression. Cells were co-transfected with the IGF-1 3′-UTR luciferase reporter construct along with either miRNA mimetics or a control non-targeting sequence. MiR-126-5p, miR-194-2-3p, and miR-379, but not Let-7d, significantly silenced the IGF-1 reporter (Figure 5A). We next assessed the influence of terminal uridine additions on the silencing activity of these miRNAs by comparing the effects on the IGF-1 reporter of unmodified miRNA mimetics to those with 2 uridines added onto the 3′ end. The uridylation of miR-126-5p or miR-379 significantly diminished IGF-1 silencing by these miRNAs, while uridylation of miR-194-2-3p had no effect (Figure 5B). These data demonstrate that uridylation of specific miRNA species may influence silencing. Interestingly, varying the length of the terminal uridine tail, to reflect the different forms observed for each of these miRNAs in our deep sequencing datasets, had minimal impact for both miR-126-5p and miR-379 (Figure 5C–5D), demonstrating that even a single uridine is sufficient to mitigate silencing by these miRNAs. Terminal uridylation did not completely eliminate silencing effects, suggesting that these end modifications provide tuning ability rather than a binary on-off switch. The effects of adding uridine(s) to the 3′ terminus were modest in comparison to the effects of altering bases in the seed region of the 5′ terminus, which completely eliminated repression of the IGF-1 3′-UTR reporter (Figure 5E), indicating that the effects described here reflect a scaling of canonical miRNA activity. Interestingly, when 2 miRNAs targeting the IGF-1 3′-UTR were simultaneously co-transfected, silencing was minimally influenced if only 1 of the 2 was uridylated, but it was very effectively attenuated when both were uridylated (Figure 5F). These data indicate that uridylation events have cumulative effects across the set of miRNAs targeting a given 3′-UTR. The combination of such effects provides a wide dynamic range over which expression can be tuned.

### IGF-1 expression is compromised by Zcchc11 deficiency *in vivo*

**Figure 6:**
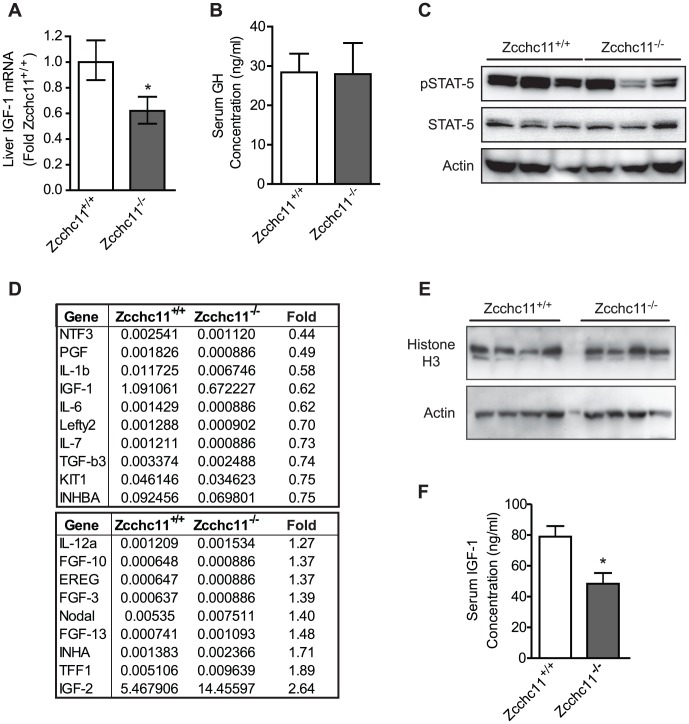
Zcchc11 deficiency decreases IGF-1 expression *in vivo*. (A) IGF-1 mRNA, measured by qRT-PCR, was decreased by Zcchc11 deficiency in 8-day-old livers. *p<0.05 *vs* Zcchc11^+/+^ by paired student's t-test. (B) Growth hormone (GH) in serum, measured by ELISA, was unaffected by genotype in 8-day-old mice, based on no significant difference using student's t-test. (C) STAT5 content and phosphorylation, as detected by immunoblot, were unaffected by Zcchc11 deficiency in the 8-day-old liver. (D) Growth factor PCR array of the livers from 8-day-old mice revealed IGF-1 to be the only transcript in this set to be substantially expressed and diminished by Zcchc11 deficiency. Numerical values for each genotype indicate dCT of transcript expression normalized against the mean expression of three separate housekeeping genes. The “fold” column represents the fold-change expression in Zcchc11^−/−^ compared to Zcchc11^+/+^ livers. (E) Histone H3 content, as detected by immunoblot, was unaffected by Zcchc11 deficiency in the 8-day-old liver. (F) IGF-1 protein was decreased in the serum of 8-day-old Zcchc11^−/−^ mice. *p<0.05 *vs* Zcchc11^+/+^ by student's t-test.

The above results, demonstrating that Zcchc11 contributes to the uridylation of miRNAs which target IGF-1 and that miRNA uridylation relieves silencing to enhance IGF-1 expression, suggested the possibility that the decreased size and survival of Zcchc11-deificient mice may be associated with decreased expression of IGF-1 *in vivo*. Supporting this hypothesis, Zcchc11 deficiency reduced hepatic IGF-1 expression to approximately half of wild type levels (Figure 6A). To differentiate regulation of the IGF-1 transcript in the liver from upstream signals, we examined STAT5 phosphorylation in the liver and growth hormone (GH) in the blood. Neither was affected by Zcchc11 deficiency (Figure 6B–6C), suggesting a local hepatocyte role for this enzyme in regulating IGF-1 mRNA. To test whether IGF-1 expression was selectively enhanced or whether many diverse growth factors were dependent upon Zcchc11 in neonatal mouse livers, we performed a PCR array for mouse growth factor transcripts. Only IGF-1 was strongly expressed and diminished by Zcchc11 deficiency in these livers (Figure 6D). The impact of Zcchc11 deficiency on IGF-1 was comparable to its impact on IL-6, which was previously demonstrated to depend on Zcchc11 [3]. These data reveal that Zcchc11 effects are transcript-specific rather than transcriptome-wide, and that IGF-1 is a particular target of Zcchc11 regulation. We also measured the expression of histone H3 in these livers, since this protein contributes to cell proliferation and is enhanced by Zcchc11 in some but not other cell lines [18], . There was no effect of Zcchc11 deficiency on histone H3 content in the young mouse liver (Figure 6E). Reflecting the changes in liver transcript, Zcchc11 deficiency reduced circulating IGF-1 concentrations to about half of wild type levels (Figure 6F), suggesting that diminished IGF-1 could contribute to systemic phenotypes. We conclude that Zcchc11 is essential to facilitating IGF-1 expression during neonatal periods.

## Discussion

The present communication reports, to our knowledge, the first studies of Zcchc11 *in vivo*. We find that Zcchc11 is widely expressed across multiple tissues shortly after birth and that in neonatal mice Zcchc11 deficiency results in a failure to thrive, associated with diminished IGF-1 expression. Mice with a complete IGF-1 deficiency have perinatal lethality and decreased growth rates [15], [16] consistent with, but more severe than, the phenotypes observed in the Zcchc11-deficient mice. Like the Zcchc11-deficient mice, genetic engineering that reduces but does not eliminate IGF-1 signaling causes proportionally decreased growth [20], [21]. Zcchc11 deficiency results in decreased uridylation of miRNAs in the liver, including miRNAs that target the IGF-1 3′-UTR. Gene expression tied to this 3′-UTR is enhanced by the uridylation of miRNAs or the increased expression of Zcchc11. Altogether, we interpret these results as supporting a model in which the uridylation of miRNAs by Zcchc11 in the neonatal liver is essential for optimal IGF-1 expression and its promotion of growth and survival through the early postnatal period. However, Zcchc11 is a large and multifunctional protein, and we recognize that the abilities of Zcchc11 to uridylate other substrates [19] and to exert uridylation-independent activities [18] may additionally contribute to the complex phenotypes of Zcchc11-deficient mice.

The decreased growth rate of Zcchc11-deficient mice is complementary to the increased growth rate observed in Lin28a-overexpressing transgenic mice [22]. Knockdown of Lin28 and Zcchc11 in cell lines results in similar phenotypes, and these proteins physically interact [6], [7], [8]. These similarities support the concept that Zcchc11 and Lin28 are involved in overlapping pathways *in vivo*. However, the suggestion that Zcchc11-Lin28 interactions may be essential for embryonic stem cell maintenance [6], [7], [8] is not supported by our observation that Zcchc11 deficiency does not impact growth or survival during embryogenesis. Like Zcchc11, Lin28 proteins are particularly expressed in the tissues of young mice [23]. It will be of great interest to learn whether mice with deficiencies in Lin28a, Lin28b or both have phenotypes involving perinatal lethality and decreased growth, as observed with the Zcchc11-deficient mice.

Untemplated uridines and adenines on mature miRNAs are consistent findings in deep sequencing analyses [5], [24], but the mechanisms and significance of such terminal additions have been difficult to discern. Previous *in vitro* studies had suggested that Zcchc11 is one of several enzymes capable of end-modifying mature miRNAs and that miRNA sequence variety might regulate transcript expression [3], [12]. The only other mouse model of PAP mutation, mice deficient in PAPD4/GLD-2, has no reported growth or survival phenotype [4], [25]. Along with our data that other PAPs were expressed in the neonatal livers of the Zcchc11-deficient mice, these findings conclusively demonstrate that the different PAPs have unique and non-overlapping roles *in vivo*. Unlike GLD-2, Zcchc11 is critical for thriving through the neonatal period, likely due in part to its ability to enhance hepatic IGF-1 expression.

In embryonic stem cell lines, Zcchc11 knockdown increases let-7 levels, due to Zcchc11-mediated uridylation of precursors [6], [7]. The Zcchc11-deficient mice allowed the examination of miRNA regulation in primary cells of living animals, and they revealed that Zcchc11 is not an essential determinant of mature let-7 or any mature miRNA quantity in the neonatal liver. Furthermore, insertional mutagenesis of Zcchc11 did not increase let-7 quantities in primary embryonic stem cells derived from these mice. The relationships among these terminal uridyltransferases (Zcchc11 and Zcchc6), Lin28 proteins (a and b), and let-7 miRNAs in embryonic stem cells are complex and dynamic [26], [27]. The present data show that Zcchc11 is not an absolute requirement for stem cell maintenance or low levels of let-7. There may be other conditions in which precursor uridylation by Zcchc11 is essential to regulating let-7, such as perhaps early embryogenesis when Lin28a is especially active. This mouse model will serve as a useful resource for determining if and when this uridyltransferase enzyme may influence miRNA biogenesis or content.

In contrast to the unchanged abundance of miRNAs in the liver, mature miRNA lengths and sequences were altered by Zcchc11 deficiency. Mature miRNAs in the liver were longer and more likely to end in uridine, including untemplated uridines, when Zcchc11 was present. Thus, our data show that Zcchc11 functions to uridylate mature miRNAs *in vivo*.

In addition to providing unprecedented evidence of an enzyme actively uridylating the 3′ terminus of miRNAs *in vivo*, these mice yield new insights into the scope of Zcchc11 modification of the miRNome. Rather than targeting only one individual miRNA, as has been previously documented for Zcchc11 and other PAPs [3], [4], [5], we show here that Zcchc11 targets the 3′ terminus of multiple miRNAs. This broad substrate repertoire dramatically increases the potential targeting power of Zcchc11. Importantly, most of the end-modifications observed varied by a small number of terminal nucleotides, and we observed that even a single uridine addition was sufficient to alleviate silencing activity. Such mono-uridylation by Zcchc11 appears to distinguish the effect of Zcchc11 on mature miRNAs from that described for pre-miRNAs, which is processive and results in a string of uridines being added [6], [28]. Our data further expand our understanding of the molecular implications of miRNA uridylation by demonstrating that coordination of miRNA uridylation events across a 3′-UTR have combinatorial effects. The ability to adjust the silencing activity of many miRNAs targeting one transcript provides a wide dynamic range for enhancing gene expression. The exonuclease Nibbler was recently identified as capable of shortening miRNAs by removing terminal nucleotides [29], [30]. Such enzymes may counter-balance the nucleotidyltransferase activities of PAPs like Zcchc11. The abundance and remarkable stability of miRNAs suggest that mechanisms regulating miRNA activity are crucial, but they are only beginning to be elucidated [31].

We propose that the miRNA 3′ terminus functions as a critical regulatory node that is remodeled by diverse enzymes to adjust miRNA silencing and tune gene expression. The present results support this nascent paradigm by demonstrating essential *in vivo* roles of Zcchc11 in miRNome remodeling and postnatal development. Zcchc11 mediates mature miRNA uridylation, facilitates hepatic IGF-1 expression, and enhances growth and survival through the neonatal period.

## Materials and Methods

### Zcchc11-deficient mouse model

A mouse embryonic stem (ES) cell line (RRR277) containing a gene-trap insertion in the *Zcchc11* gene was obtained from BayGenomics at the Mutant Mouse Regional Resource Center at University of California-Davis [10]. C57BL/6 blastocysts were microinjected with mutant ES cells to create chimeric mice that were subsequently backcrossed onto a C57BL/6 genetic background for at least 10 generations. The gene-trap genomic insertion site was located within intron 4 of *Zcchc11*, generating a fusion protein containing the first 314 amino acids of Zcchc11 joined in frame with the β-galactosidase reporter. All known conserved protein motifs and domains in Zcchc11 are downstream of this site. The mutant allele was detected by genomic PCR using the primers listed in Table S2. All murine studies were performed under approval of the Boston University School of Medicine IACUC.

### Embryonic stem cell derivation

Eight-cell stage mouse embryos were collected in M2 medium (Millipore) from superovulated *Zcchc11* ^+/+^, *Zcchc11* ^+/−^, or *Zcchc11* ^−/−^ females mated with *Zcchc11* ^+/+^ or *Zcchc11* ^+/−^ males. Embryos were cultured to the early blastocyst stage in KSOM (Millipore) supplemented with 2i (1 µM PD0325901 and 3 µM CHIR99021 (Cayman Chemical)), followed by 48 hours of culture in Neurobasal medium supplemented with N2, B27 (Invitrogen) 2i and Leukemia Inhibitory Factor (LIF, Millipore) at 37°C, under 5% CO_2_. The resulting expanded blastocysts were cultured on laminin (Sigma, 10 µg/ml) coated tissue culture plastic in N2B27+2i+LIF until the blastocysts had attached and outgrowths were visible (3–4 days). These ES outgrowths were recovered by mouth pipette, disaggregated to single cells with 0.25% Trypsin (Invitrogen) and plated on laminin-coated tissue culture plastic to establish embryonic stem cell lines. Cell line genotype was determined by amplifying wild type and mutant products of *Zcchc11* alleles.

### Cell line transfections

H1299 cells were plated at 2.5×10^6^ cells/well in 6-well plates and transfected with 0.5 µg reporter construct, 0.25 µg phRLTK control reporter (Promega) and 200 nM miRNA mimetic using 4 µl/well Lipofectamine 2000 (Invitrogen). EGFP or Zcchc11 constructs were transfected at 3 µg/well. Luciferase was measured using the Dual Luciferase Reporter Assay System (Promega). Mimetics were duplexed siRNA (Dharmacon); sequences are presented in Table S2.

### RNA and protein assays

Liver growth factors were measured by PCR Array (SA Biosciences) using 1 µg RNA pooled from 4 Zcchc11^+/+^ or 4 Zcchc11^−/−^ mice. IGF-1 and 18S rRNA qRT-PCR was performed using the TaqMan RNA-to-Ct kit (Applied Biosystems) with primers and probes shown in Table S2. Small RNAs were reverse transcribed using the TaqMan miRNA assay system (Applied Biosystems). Northern blot analysis was performed on 15 µg of total RNA electrophoresed through a 1% agarose-formaldehyde gel and immobilized on a BrightStar-Plus nylon membrane. For probe creation, IGF-1 cDNA (Open Biosystems) was digested out of a pCMV-Sport6 vector using XbaI and SalI restriction enzymes (NEB). GAPDH probes were purchased (SA Biosciences). Antibodies were obtained from Cell Signaling Technologies except goat anti-Zcchc11 (ProSci), Rabbit anti-Goat (R&D), Rabbit anti-GLD-2 (Abgent), and Rabbit anti-PAPD5 (Genetex). Whole blood was collected from the hepatic vein of 10-week old mice or by cardiac puncture from 8-day-old litters of Zcchc11^+/−^ breeding pairs; serum was separated and components were measured using mouse IGF-1 and GH ELISA Kits (R&D Systems).

### Cloning and plasmid construction

All Zcchc11 overexpression studies were performed using the same plasmid backbone as the control Enhanced GFP (pEGFP-N, Clontech). Creation of the Zcchc11 and DADA mutant plasmids has been previously described [3]. The N-terminal region of Zcchc11 was PCR amplified from the Zcchc11 plasmids using Phusion High Fidelity DNA Polymerase (NEB) with the primers indicated in Table S2. The resulting product was ligated between *NotI* and *BsrGI* sites in the full length Zcchc11 plasmid. The full-length IGF-1 3′ UTR (Accession # NM_001111274.1) was amplified from a cDNA library of whole liver RNA from 8 day-old C57BL/6 mice with Herculase II DNA polymerase (Agilent) using primers shown in Table S2. The resulting band was ligated between *SacI* and *MluI* restriction sites into the pMir-Report expression vector (Ambion). All plasmid constructs were sequenced.

### Small RNA cloning and deep sequencing

Livers from 8 day-old mice were stored in RNA later (Qiagen). Half of each liver was homogenized in BioPure RNA isolation reagent (BiooScientific) with 0.5 mm zirconium oxide beads using a bullet blender (Next Advance). RNA <30 nt long was purified from phenol-chloroform extracted RNA using a FlashPAGE fractionator (Ambion). Small RNA library creation was performed using an adapted Illumina small RNA sample prep v1.5.0. Briefly, small RNA samples were adaptor ligated with one of four different 3′ adaptors using T4 RNA Ligase 2 (NEB) to allow multiplex sequencing of Zcchc11^+/+^ and Zcchc11^−/−^ samples. A conserved 5′ adaptor was added using truncated T4 RNA Ligase 2. Samples were gel extracted on a 10% TBE-Urea gel to remove adaptor only ligation products. cDNA was created using SuperScript III Reverse Transcriptase (Invitrogen) then PCR amplified with GoTaq DNA Polymerase (Promega). Adaptor and primer sequences are shown in Table S2. The resulting library was sequenced on an Illumina Genome Analyzer IIx. In total, three libraries were created each containing RNA from two Zcchc11^+/+^ and two Zcchc11^−/−^ sex matched littermates. Libraries were uploaded to the NCBI Sequence Reads Archive (Accession Number: SRA059070).

### Analysis of deep sequencing data

All library analyses were performed using the Genomic Workbench software platform (CLC Biosystems). First, sequences were sorted by adaptor barcode and perfectly matched adaptor sequences were trimmed while ambiguous/unrecognizable adaptor sequences were discarded. The samples were then grouped, counted and aligned to v16.0 of miRBase [13], [14] allowing no more than 2 internal mismatches and no more than 5 at the 3′ end of the sequence. For identification of sequence modifications the libraries were aligned to the mouse genome annotated with v9.0 of the mouse database from the UCSC genome browser. SNP detection was performed using the neighborhood quality score algorithm [32] to identify sequence variants occurring at greater than one per 1,000 sequences. Full genome alignment was used to empower the use of quality scores (average quality score of 15, minimum central quality of 20, with a window length of 5) for SNP detection. SNP position alignment data was exported for further analysis and adenylation/uridylation was defined as A or T variants occurring at the position one nucleotide beyond the published 3′ terminal end of the miRNA. Unambiguous uridylation events were defined as substitutions of a non-genomic U for any other nucleotide at the 3′ terminal end of miRNA.

## Supporting Information

Figure S1 Engineering and postnatal development of Zcchc11^−/−^ mice. (A) PCR confirmation of β-gal insertion into the Zcchc11 gene. (B) RT-PCR confirmation of β- gal incorportation into the Zcchc11 ORF. (C) Overall body weight of male and female wild type and Zcchc11 deficient mice through the first 20 weeks of life. *p<0.05 by two-way ANOVA. (D) H & E staining of the lung, brain heart, liver spleen and kidney from adult Zcchc11^+/+^ and Zcchc11^−/−^ mice.
(EPS)

Figure S2 Significant miRNA 3′ end modification in the livers of 8-day-old mice. Levels at which (A) adenosine and (B) uridine nucleotides were found at the 3′ terminal end of miRNAs from the livers of 8 day old control mice. The 40 most highly modified sequences are shown. Error bars indicate standard error from three separate libraries.
(EPS)

Table S1 Overview of library adaptor trimming and alignment.
(PDF)

Table S2 Oligonucleotide sequences used in molecular analyses.
(PDF)
