# Supplementary material for: Zcchc11 Uridylates Mature miRNAs to Enhance Neonatal IGF-1 Expression, Growth, and Survival
Source: PLoS Genet. 2012 Nov 29;8(11):e1003105. doi: 10.1371/journal.pgen.1003105 (PMC3510031; doi:10.1371/journal.pgen.1003105)
Supplement: Table S1 — Overview of library adaptor trimming and alignment. (PDF) [file pgen.1003105.s003.pdf]

Supplementary Table 1: Overview of sequences successfully trimmed of sequencing adpators and aligned to miRBase for each library. All percentages represent the percent of all sequences for that library.

	Library 1		Library 2		Library 3		Total	
	WT	KO	WT	KO	WT	KO	WT	KO
Total Sequences	6,982,747	20,582,541	20,811,654	10,164,600	13,779,581	13,323,920	41,573,982	44,071,061
Number Trimmed	6,656,219	19,732,400	18,754,122	8,917,325	12,891,049	12,652,294	38,301,390	41,302,019
Percent Trimmed	95.3%	95.9%	90.1%	87.7%	93.6%	95.0%	92.1%	93.7%
Average Length	21.9	22.0	22.7	22.6	22.1	22.0	22.2	22.2
Number Aligned*	5,256,878	17,034,524	15,436,133	7,186,297	11,929,405	10,685,924	32,622,416	34,906,745
Percent Aligned	75.3%	82.8%	74.2%	70.7%	86.6%	80.2%	78.7%	77.9%

* Aligned to miRBase v16.
