# Supplementary material for: Zcchc11 Uridylates Mature miRNAs to Enhance Neonatal IGF-1 Expression, Growth, and Survival
Source: PLoS Genet. 2012 Nov 29;8(11):e1003105. doi: 10.1371/journal.pgen.1003105 (PMC3510031; doi:10.1371/journal.pgen.1003105)
Supplement: Table S2 — Oligonucleotide sequences used in molecular analyses. (PDF) [file pgen.1003105.s004.pdf]

Supplementary Table 2: Oligo sequences.

Name	Sequence (5' - 3')	Notes
**Genotyping**		
Zcchc11 - F	TCTGCCACTTTTGTTACCTCTTTCTG	Zcchc11$^{+/+}$ genotyping forward
Zcchc11 - R	CTACAGCCTCAATAAATGATATCCTTTAATAAG	Zcchc11$^{+/+}$ genotyping reverse
β-gal - F	CAAATGGCGATTACCGTTGA	Zcchc11$^{-/-}$ genotyping forward
β-gal - R	TGCCCAGTCATAGCCGAATA	Zcchc11$^{-/-}$ genotyping reverse
**miRNA Mimetics (RNA)**		
Control	siControl #4	Non-targeting siRNA
Let-7d	AGAGGUAGUAGGUUGCAUAGUU	Let-7d mimetic
miR-126	CAUUAUUACUUUUGGUACGCG	miR-126 mimetic
miR-126U	CAUUAUUACUUUUGGUACGCGU	Uridylated mir-126 mimetic
miR-126UU	CAUUAUUACUUUUGGUACGCGUU	Double Urid. miR-126 mimetic
mir-126mut	CACGCGUACUUUUGGUACGCG	Mutated miR-126 mimetic
miR-194-2*	CCAGUGGGGCUGCUGUUAUCUG	miR-194-2* mimetic
miR-194-2*UU	CCAGUGGGGCUGCUGUUAUCUGUU	Uridylated miR-194-2* mimetic
miR-379	UGGUAGACUAUGGAACGUAGG	miR-279 mimetic
miR-379U	UGGUAGACUAUGGAACGUAGGU	Uridylated miR-379 mimetic
miR-379UU	UGGUAGACUAUGGAACGUAGGUU	Double urid. miR-379 mimetic
miR-379UUUU	UGGUAGACUAUGGAACGUAGGUUUU	Quadruple Urid. miR-379 mimetic
**Northern Blotting**		
cLet-7a	AACTATACAACCTACTACCTCA	Complement of Let-7a
cU6	GCCATGCTAATCTTCTCTGTATC	Complement of U6 snRNA
**rtPCR**		
Zcchc6-F	CGCTTACACTCTTATGGTGCTATACTT	Zcchc6 qRT-PCR forward primer
Zcchc6-R	CTGGTTTCTTTTCACCTTTGATATCT	Zcchc6 qRT-PCR reverse primer
Zcchc6 Probe	AGGTCTCCACCTGTCATC	Zcchc6 qRT-PCR probe
Igf-1 - F	TTCTACCTGGCGCTCTGCTT	Igf-1 qRT-PCR forward primer
Igf-1 - R	CTCGGTCCACACACGAACT	Igf-1 qRT-PCR reverse primer
Igf-1 Probe	AGCATCCACCAGCTCAG	Igf-1 qRT-PCR probe
18S - F	ATTCGAACGTCTGCCCTATCA	Control qRT-PCR forward primer
18S - R	GTCACCCGTGGTCACCATG	Control qRT-PCR reverse primer
18S - probe	TCGATGGTAGTCGCCGTGCC	Control qRT-PCR prove
**Cloning Primers**		
Zcchc11-N - F	CTACAGCGTCAGATTGGAAAGAGTC	Zcchc11 N terminal forward
Zcchc11-N - R	CCCGCGGCCGCTTACTTGTCATCGTCGTCCTTGTAA TCCCCAATGCAAATACACTGGCT	Zcchc11 N reverse + NotI site
Igf-1 F	GGGGAGCTCTAGAGGAAGTGCAGGAAACAAGACC	Igf-1 3' UTR forward + SacI site
Igf-1 R	CCCACGCGTACCAGTTAATCAAACATGATTAATTTTAATG	Igf-1 3' UTR reverse + Mlu site
**Deep Sequencing**		
3' Barcode 1†	rApp/AAGTATCGTATGCCGTCTTCTGCTTG/ddC	3' Seqeucing Adaptor Seq. 1
3' Barcode 2†	rApp/ATCCTTCGTATGCCGTCTTCTGCTTG/ddC	3' Sequecing Adaptor Seq. 2
3' Barcode 3†	rApp/AGAGGTCGTATGCCGTCTTCTGCTTG/ddC	3' Sequecing Adaptor Seq. 3
3' Barcode 4†	rApp/TAGGTTCGTATGCCGTCTTCTGCTTG/ddC	3' Sequecing Adaptor Seq. 4
5' Adapter*	GUUCAGAGUUCUACAGUCCGACGAUC	5' Sequecning Adaptor
rtPrimer*	CAAGCAGAAGACGGCATACGA	Deep sequencing rtPCR primer
Seq. Reverse*	CAAGCAGAAGACGGCATACGA	Deep sequencing reverse primer
Seq. Forward*	ATGATACGGCGACCACCGACAGGTTCAGAGTTCTACA GTCCGA	Deep sequencing forward primer (Includes extension for flow cell)
